# Development and validation of a nomogram predicting the risk of necrosis in fingertip replantation: a single-center retrospective study

**DOI:** 10.3389/fsurg.2026.1926687

**Published:** 2026-09-15

**Authors:** Yadong Li, Xinwei Hou, Shuai Dong, Yan Zhang, Zhongliang Wang, Jihui Ju, Guangzhe Jin

**Affiliations:** 1 Department of Hand Surgery, Suzhou Ruihua Orthopedic Hospital, Suzhou, Jiangsu, China; 2 Suzhou Medical College of Soochow University, Suzhou, Jiangsu, China

**Keywords:** fingertip replantation, necrosis, neutrophil percentage, nomogram, prediction model

## Abstract

**Background:**

Necrosis remains an important complication after fingertip replantation. We aimed to develop and internally validate a model for estimating necrosis risk using clinical, procedural, and early-postoperative information.

**Methods:**

We retrospectively analyzed 1,344 patients who underwent replantation of 1,497 fingertips at a single center between August 2018 and August 2023. The patient was the unit of analysis. Patients were divided by outcome-stratified random sampling (seed 2222) into a derivation cohort (*n* = 942; 70%) and a split-sample internal validation cohort (*n* = 402; 30%). Candidate predictors were reduced by 10-fold cross-validated least absolute shrinkage and selection operator regression. A multivariable logistic regression model was specified using six predictors available during the early postoperative course. Discrimination, calibration, Brier score, and decision-curve analysis were evaluated.

**Results:**

Necrosis occurred in 115 patients (12.2%) in the derivation cohort and 48 (11.9%) in the internal validation cohort. The model retained surgeon experience, platelet count (PLT), neutrophil percentage (NEU%), D-dimer, hypertension, and vascular crisis. The area under the receiver operating characteristic curve was 0.953 (95% CI, 0.935–0.968) in the derivation cohort and 0.974 (95% CI, 0.958–0.987) in the internal validation cohort. Corresponding Brier scores were 0.051 and 0.042. In the internal validation cohort, the calibration intercept was −0.338 and the calibration slope was 0.759; the Hosmer–Lemeshow test yielded *P* = 0.729. Decision-curve analysis suggested potential net benefit across clinically plausible threshold probabilities, but external validation is required.

**Conclusion:**

This split-sample internally validated nomogram estimates necrosis risk after fingertip replantation once early-postoperative laboratory results and vascular-crisis status are available. The observed discrimination should be interpreted alongside calibration shrinkage, possible overfitting, and the absence of external validation.

## 1 Background

As a rapidly developing manufacturing country, China has seen a significant increase in hand injuries, particularly fingertip amputations, which are common in hand trauma cases. Severe fingertip injury was once considered a contraindication for replantation, with most surgeons opting for amputation (1, 2) or the use of flaps (3, 4). However, the influence of traditional Chinese concepts coupled with the widespread availability of social health insurance, Chinese patients has led to elevated expectations among Chinese patients regarding the preservation of their fingers, resulting in a significantly higher rate of fingertip replantation in China compared with other countries. With the development of microsurgical techniques, the success rate of fingertip replantation in most Chinese hospitals has exceeded 80%. However, fingertip replantation still faces numerous challenges, and some patients experience failure postoperatively. Tissue necrosis not only affects the psychological health of patients but can also have long-term consequences for the appearance and functionality of the hand (5).

Previous studies have identified predictors of digit-replantation failure and have proposed prediction models, including models based on large retrospective or multicenter cohorts (5–8). The present study differs by focusing on distal fingertip replantation and integrating procedural characteristics with routinely available early-postoperative laboratory markers and vascular-crisis status. It is therefore intended as an early-postoperative, rather than preoperative, risk-stratification tool. This incremental contribution and its potential clinical use require confirmation through external validation.

## 2 Patients and methods

### 2.1 Study design and patient population

We retrospectively screened 1,344 patient records covering 1,497 replanted fingertips treated at our hospital between August 2018 and August 2023. No patient was excluded for missing predictor or outcome data. The patient was the unit of analysis: each patient contributed one record and one binary patient-level outcome, including patients with multiple replanted fingertips. Patients were divided by outcome-stratified random sampling (seed 2222) into a derivation cohort (*n* = 942; 70%) and a split-sample internal validation cohort (*n* = 402; 30%). Inclusion criteria were (i) fingertip amputation at or distal to the distal interphalangeal joint and (ii) age >18 years. Exclusion criteria were (i) refusal of follow-up or unavailable essential predictor/outcome data; (ii) absence of vascular or nerve anastomosis; (iii) mental illness or communication disorders; and (iv) vascular injury in the ipsilateral arm or forearm. Data were anonymized. The institutional ethics committee approved the study and waived informed consent. The participant flow is shown in Figure 1.

**Figure 1:**
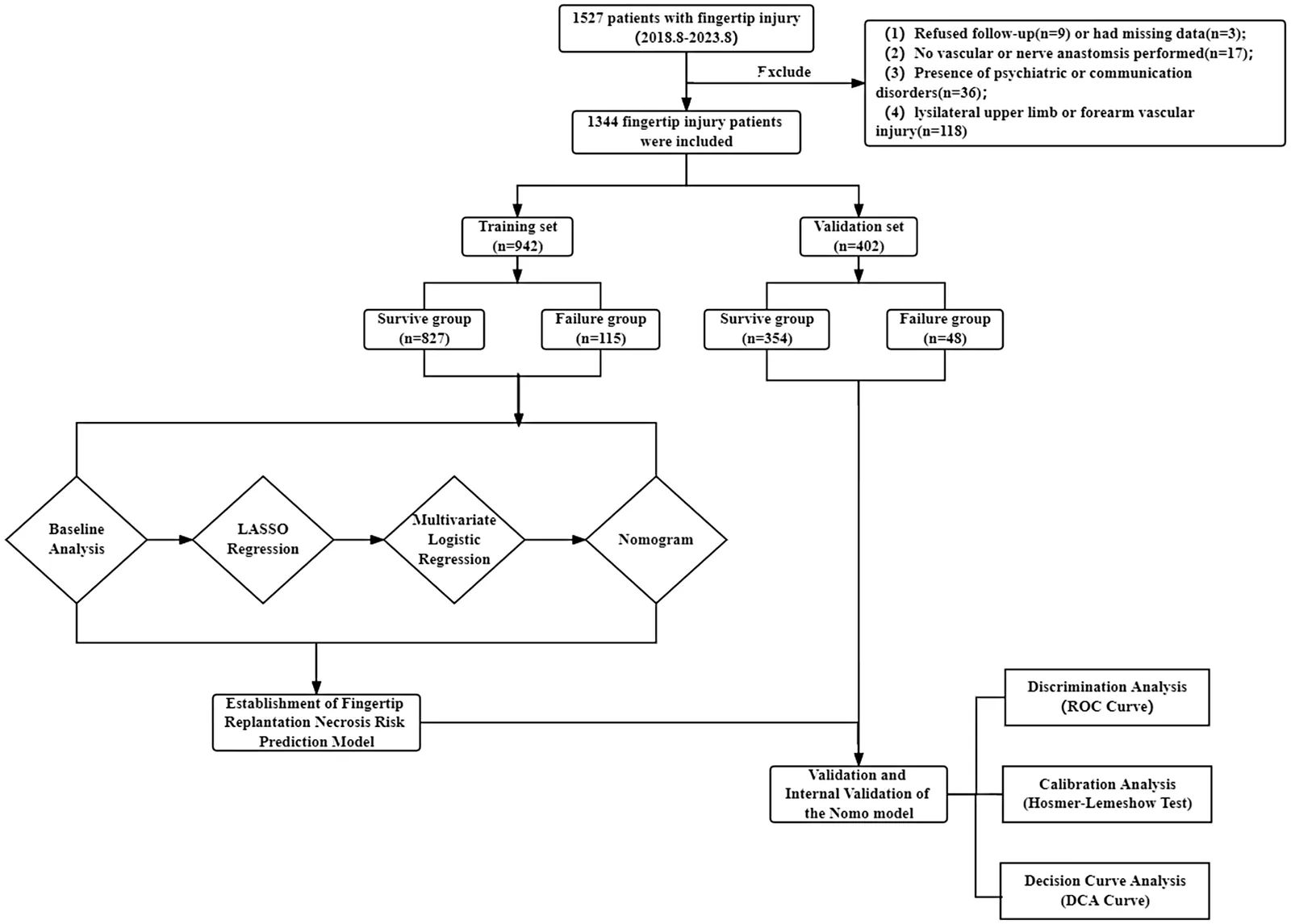
Study flowchart. Flow diagram of patient inclusion, cohort allocation, variable selection, model development, and validation.

### 2.2 Potential prognostic factors

Demographic data such as age, sex, smoking status (smoker or non-smoker), and comorbidities (e.g., hypertension, diabetes) were self-reported. Clinical risk factors were derived from medical records. Preoperative risk factors included injury mechanism, which finger was injured, injury level, number of amputated fingers, preservation method of amputated fingers, presence of other injuries, degree of amputation (complete or incomplete), bone and joint injury, nerve injury, tendon injury, and nail bed or dorsal skin injury.

Intraoperative risk factors included ischemia time (the time elapsed between the restoration of blood flow and the reported injury time), duration of surgery, surgeon experience (specialist was defined as having performed more than 50 finger replantation surgeries, while trainee was defined as having performed 49 or fewer finger replantations), vein graft usage, number of arteries repaired, number of veins repaired, arterial to venous ratio, arterial injury severity, and whether flap coverage was used.

Postoperative candidate predictors included PLT (×10⁹/L), NEU% (percentage points), hemoglobin (g/L), albumin (g/L), D-dimer (mg/L), and vascular crisis. Laboratory measurements were obtained 3–5 days after replantation. Vascular crisis was defined as a clinically documented arterial or venous perfusion disturbance after surgery and before determination of the final replantation outcome: arterial crisis comprised ischemia due to arterial-anastomosis occlusion or inadequate inflow, whereas venous crisis comprised congestion due to venous-anastomosis occlusion or inadequate outflow. The model is intended for use during the early postoperative course, after these laboratory results and vascular-crisis status have become available; it is not a preoperative model.

### 2.3 Outcome definition and assessment

The binary outcome was complete necrosis/replantation failure documented in the medical record on the basis of irreversible loss of perfusion and tissue viability. Partial superficial necrosis without complete replantation failure was not counted as an event. Outcome status was abstracted from the final documented clinical assessment during available follow-up; no uniform minimum follow-up interval was imposed. Assessments were made by the treating hand-surgery team, who were not blinded to clinical and laboratory information. These features may introduce ascertainment bias.

### 2.4 Statistical analysis

Continuous variables are reported as mean ± standard deviation or median (interquartile range), as appropriate. Distributional assumptions were evaluated before between-group comparisons; independent-samples *t* tests or Mann–Whitney U tests were used for continuous variables, and chi-square or Fisher exact tests were used for categorical variables. Baseline characteristics were compared between the derivation and internal validation cohorts.

Candidate variables were chosen from demographic, injury-related, procedural, and early-postoperative factors available in the dataset. The resulting dataset contained no missing predictor or outcome values; therefore, neither case deletion nor imputation was required. Least absolute shrinkage and selection operator (LASSO) logistic regression with 10-fold cross-validation was used for variable reduction. The one-standard-error criterion (lambda.1se = 0.01601135; log [lambda.1se] = −4.134) retained nine candidate variables. Six clinically interpretable variables were retained in the multivariable logistic model and nomogram. Binary predictors were indicator-coded; any arterial or venous crisis was coded as vascular crisis present. PLT was scaled per 10 × 10⁹/L, NEU% per percentage point, and D-dimer per 1 mg/L. Multicollinearity was assessed using variance inflation factors (maximum VIF = 1.09). Model discrimination was quantified by the area under the receiver operating characteristic curve (AUC) with bootstrap 95% confidence intervals. Calibration was assessed using plots, calibration intercept and slope, Brier score, and the Hosmer–Lemeshow test. Decision-curve analysis was restricted to clinically interpretable thresholds. Reporting was revised with reference to the TRIPOD framework.

## 3 Results

### 3.1 Comparison of the derivation and internal validation cohorts

The analysis cohort comprised 1,344 patients, of whom 163 (12.1%) experienced necrosis. Outcome-stratified random sampling assigned 942 patients to the derivation cohort (115 necrosis events) and 402 to the split-sample internal validation cohort (48 events). Baseline characteristics were broadly similar between cohorts (Table 1).

**Table 1:** Complete baseline characteristics of the derivation and internal validation cohorts.

Variable	Derivation (*n* = 942)	Internal validation (*n* = 402)	*P* value
Necrosis			0.963
No	827 (87.8%)	354 (88.1%)	
Yes	115 (12.2%)	48 (11.9%)	
Sex			0.018
Male	732 (77.7%)	336 (83.6%)	
Female	210 (22.3%)	66 (16.4%)	
Age (years)	38.5 ± 14.0	40.2 ± 13.7	0.033
Ischemia time (hours)	5.1 ± 2.0	5.3 ± 2.0	0.181
Fingertip preservation			0.389
Isolated ice-bag cooling	3 (0.3%)	0 (0.0%)	
Clean dressing	744 (79.0%)	326 (81.1%)	
Room temperature/other	195 (20.7%)	76 (18.9%)	
Multiple/associated injury			0.140
No	856 (90.9%)	354 (88.1%)	
Yes	86 (9.1%)	48 (11.9%)	
Duration of surgery (hours)	2.8 ± 1.3	3.3 ± 1.6	0.000
Surgeon experience			0.003
Trainee	197 (20.9%)	115 (28.6%)	
Specialist	745 (79.1%)	287 (71.4%)	
Injured finger			0.666
Thumb	165 (17.5%)	70 (17.4%)	
Index	275 (29.2%)	100 (24.9%)	
Middle	191 (20.3%)	89 (22.1%)	
Ring	134 (14.2%)	65 (16.2%)	
Little	83 (8.8%)	35 (8.7%)	
Multiple digits	94 (10.0%)	43 (10.7%)	
Number of injured fingers			0.702
One	849 (90.1%)	360 (89.6%)	
Two	81 (8.6%)	39 (9.7%)	
Three	10 (1.1%)	2 (0.5%)	
Four	2 (0.2%)	1 (0.2%)	
Injury level			0.509
Free tissue block	72 (7.6%)	34 (8.5%)	
Distal to nail	132 (14.0%)	43 (10.7%)	
Middle nail level	205 (21.8%)	84 (20.9%)	
Nail-base level	306 (32.5%)	140 (34.8%)	
Distal interphalangeal joint	227 (24.1%)	101 (25.1%)	
Injury mechanism			0.344
Electric saw	56 (5.9%)	28 (7.0%)	
Crush	464 (49.3%)	218 (54.2%)	
Twisting	173 (18.4%)	59 (14.7%)	
Blunt impact	48 (5.1%)	17 (4.2%)	
Heat press	1 (0.1%)	0 (0.0%)	
Avulsion	16 (1.7%)	3 (0.7%)	
Sharp cut	184 (19.5%)	77 (19.2%)	
Smoking history			0.748
No	791 (84.0%)	334 (83.1%)	
Yes	151 (16.0%)	68 (16.9%)	
Hypertension			0.936
No	883 (93.7%)	378 (94.0%)	
Yes	59 (6.3%)	24 (6.0%)	
Diabetes			0.799
No	929 (98.6%)	395 (98.3%)	
Yes	13 (1.4%)	7 (1.7%)	
Vein graft			0.108
No	925 (98.2%)	400 (99.5%)	
Yes	17 (1.8%)	2 (0.5%)	
Degree of amputation			0.736
Incomplete	39 (4.1%)	19 (4.7%)	
Complete	903 (95.9%)	383 (95.3%)	
Bone injury			0.709
Comminuted fracture	343 (36.4%)	140 (34.8%)	
Intra-articular fracture	211 (22.4%)	98 (24.4%)	
Transverse fracture	388 (41.2%)	164 (40.8%)	
Nerve injury			0.216
Fingertip nerve injury	267 (28.3%)	104 (25.9%)	
Bilateral crush laceration	344 (36.5%)	167 (41.5%)	
Bilateral sharp laceration	179 (19.0%)	79 (19.7%)	
Bilateral avulsion injury	152 (16.1%)	52 (12.9%)	
Tendon injury			0.314
No tendon rupture	696 (73.9%)	294 (73.1%)	
Flexor/extensor zone I	235 (24.9%)	107 (26.6%)	
Flexor/extensor zone II	7 (0.7%)	0 (0.0%)	
Flexor/extensor zone V	4 (0.4%)	1 (0.2%)	
Number of arteries repaired			0.459
One	116 (12.3%)	46 (11.4%)	
Two	747 (79.3%)	314 (78.1%)	
Three or more	79 (8.4%)	42 (10.4%)	
Number of veins repaired			0.789
One	166 (17.6%)	78 (19.4%)	
Two	283 (30.0%)	109 (27.1%)	
Three	152 (16.1%)	64 (15.9%)	
Four	220 (23.4%)	94 (23.4%)	
Five or more	121 (12.8%)	57 (14.2%)	
Artery-to-vein repair ratio			0.534
1:0	9 (1.0%)	2 (0.5%)	
1:1	77 (8.2%)	37 (9.2%)	
1:2	53 (5.6%)	15 (3.7%)	
2:1	85 (9.0%)	43 (10.7%)	
2:2	272 (28.9%)	111 (27.6%)	
≥2:3	446 (47.3%)	194 (48.3%)	
Arterial injury			0.050
Fingertip artery transection	265 (28.1%)	97 (24.1%)	
Bilateral digital-artery crush injury	338 (35.9%)	171 (42.5%)	
Bilateral digital-artery sharp transection	179 (19.0%)	83 (20.6%)	
Bilateral digital-artery avulsion	146 (15.5%)	50 (12.4%)	
Avulsion with unilateral defect >1.5 cm	2 (0.2%)	0 (0.0%)	
Avulsion with bilateral defects >1.5 cm	12 (1.3%)	1 (0.2%)	
Nail-bed/dorsal skin injury			0.636
Nail-bed laceration	397 (42.1%)	154 (38.3%)	
Nail-bed and dorsal-skin injury	501 (53.2%)	232 (57.7%)	
Crush injury	9 (1.0%)	5 (1.2%)	
Sharp/blunt laceration	23 (2.4%)	7 (1.7%)	
Avulsion injury	7 (0.7%)	3 (0.7%)	
Avulsion with partial skin defect	5 (0.5%)	1 (0.2%)	
PLT (×10⁹/L)	235.5 ± 59.9	230.4 ± 57.8	0.140
NEU% (%)	66.3 ± 10.7	67.3 ± 9.9	0.094
D-dimer (mg/L)	0.3 ± 0.4	0.4 ± 0.7	0.461
Hemoglobin (g/L)	137.5 ± 17.3	137.6 ± 17.6	0.988
Albumin (g/L)	39.1 ± 3.7	38.7 ± 3.6	0.107
Flap coverage			0.668
No	915 (97.1%)	388 (96.5%)	
Yes	27 (2.9%)	14 (3.5%)	
Vascular crisis			0.318
None	888 (94.3%)	386 (96.0%)	
Venous	6 (0.6%)	3 (0.7%)	
Arterial	48 (5.1%)	13 (3.2%)	

Values are *n* (%) or mean ± SD. *P* values are from Welch *t* tests or chi-square tests.

PLT, platelet count; NEU%, neutrophil percentage.

### 3.2 Characteristics according to necrosis outcome

In the complete cohort, the necrosis group had higher PLT, NEU%, and D-dimer values and a higher frequency of vascular crisis than the survival group. Necrosis was also more frequent in procedures performed by trainee surgeons. Selected outcome-stratified characteristics are shown in Table 2.

**Table 2:** Complete characteristics by outcome in the overall cohort.

Variable	Survival (*n* = 1181)	Necrosis (*n* = 163)	*P* value
Sex			0.062
Male	948 (80.3%)	120 (73.6%)	
Female	233 (19.7%)	43 (26.4%)	
Age (years)	38.8 ± 13.9	40.2 ± 14.5	0.269
Ischemia time (hours)	5.1 ± 1.9	5.9 ± 2.3	0.000
Fingertip preservation			0.568
Isolated ice-bag cooling	3 (0.3%)	0 (0.0%)	
Clean dressing	944 (79.9%)	126 (77.3%)	
Room temperature/other	234 (19.8%)	37 (22.7%)	
Multiple/associated injury			0.081
No	1070 (90.6%)	140 (85.9%)	
Yes	111 (9.4%)	23 (14.1%)	
Duration of surgery (hours)	2.9 ± 1.3	3.4 ± 1.7	0.001
Surgeon experience			0.000
Trainee	204 (17.3%)	108 (66.3%)	
Specialist	977 (82.7%)	55 (33.7%)	
Injured finger			0.304
Thumb	212 (18.0%)	23 (14.1%)	
Index	330 (27.9%)	45 (27.6%)	
Middle	245 (20.7%)	35 (21.5%)	
Ring	179 (15.2%)	20 (12.3%)	
Little	102 (8.6%)	16 (9.8%)	
Multiple digits	113 (9.6%)	24 (14.7%)	
Number of injured fingers			0.143
One	1070 (90.6%)	139 (85.3%)	
Two	98 (8.3%)	22 (13.5%)	
Three	10 (0.8%)	2 (1.2%)	
Four	3 (0.3%)	0 (0.0%)	
Injury level			0.000
Free tissue block	98 (8.3%)	8 (4.9%)	
Distal to nail	166 (14.1%)	9 (5.5%)	
Middle nail level	270 (22.9%)	19 (11.7%)	
Nail-base level	381 (32.3%)	65 (39.9%)	
Distal interphalangeal joint	266 (22.5%)	62 (38.0%)	
Injury mechanism			0.266
Electric saw	75 (6.4%)	9 (5.5%)	
Crush	599 (50.7%)	83 (50.9%)	
Twisting	195 (16.5%)	37 (22.7%)	
Blunt impact	62 (5.2%)	3 (1.8%)	
Heat press	1 (0.1%)	0 (0.0%)	
Avulsion	16 (1.4%)	3 (1.8%)	
Sharp cut	233 (19.7%)	28 (17.2%)	
Smoking history			0.179
No	995 (84.3%)	130 (79.8%)	
Yes	186 (15.7%)	33 (20.2%)	
Hypertension			0.000
No	1119 (94.8%)	142 (87.1%)	
Yes	62 (5.2%)	21 (12.9%)	
Diabetes			0.458
No	1165 (98.6%)	159 (97.5%)	
Yes	16 (1.4%)	4 (2.5%)	
Vein graft			0.024
No	1168 (98.9%)	157 (96.3%)	
Yes	13 (1.1%)	6 (3.7%)	
Degree of amputation			0.848
Incomplete	50 (4.2%)	8 (4.9%)	
Complete	1131 (95.8%)	155 (95.1%)	
Bone injury			0.000
Comminuted fracture	452 (38.3%)	31 (19.0%)	
Intra-articular fracture	251 (21.3%)	58 (35.6%)	
Transverse fracture	478 (40.5%)	74 (45.4%)	
Nerve injury			0.000
Fingertip nerve injury	349 (29.6%)	22 (13.5%)	
Bilateral crush laceration	439 (37.2%)	72 (44.2%)	
Bilateral sharp laceration	228 (19.3%)	30 (18.4%)	
Bilateral avulsion injury	165 (14.0%)	39 (23.9%)	
Tendon injury			0.000
No tendon rupture	893 (75.6%)	97 (59.5%)	
Flexor/extensor zone I	280 (23.7%)	62 (38.0%)	
Flexor/extensor zone II	6 (0.5%)	1 (0.6%)	
Flexor/extensor zone V	2 (0.2%)	3 (1.8%)	
Number of arteries repaired			0.034
One	147 (12.4%)	15 (9.2%)	
Two	936 (79.3%)	125 (76.7%)	
Three or more	98 (8.3%)	23 (14.1%)	
Number of veins repaired			0.091
One	223 (18.9%)	21 (12.9%)	
Two	339 (28.7%)	53 (32.5%)	
Three	182 (15.4%)	34 (20.9%)	
Four	283 (24.0%)	31 (19.0%)	
Five or more	154 (13.0%)	24 (14.7%)	
Artery-to-vein repair ratio			0.223
1:0	11 (0.9%)	0 (0.0%)	
1:1	104 (8.8%)	10 (6.1%)	
1:2	60 (5.1%)	8 (4.9%)	
2:1	117 (9.9%)	11 (6.7%)	
2:2	326 (27.6%)	57 (35.0%)	
≥2:3	563 (47.7%)	77 (47.2%)	
Arterial injury			0.000
Fingertip artery transection	339 (28.7%)	23 (14.1%)	
Bilateral digital-artery crush injury	441 (37.3%)	68 (41.7%)	
Bilateral digital-artery sharp transection	233 (19.7%)	29 (17.8%)	
Bilateral digital-artery avulsion	160 (13.5%)	36 (22.1%)	
Avulsion with unilateral defect >1.5 cm	0 (0.0%)	2 (1.2%)	
Avulsion with bilateral defects >1.5 cm	8 (0.7%)	5 (3.1%)	
Nail-bed/dorsal skin injury			0.000
Nail-bed laceration	514 (43.5%)	37 (22.7%)	
Nail-bed and dorsal-skin injury	615 (52.1%)	118 (72.4%)	
Crush injury	12 (1.0%)	2 (1.2%)	
Sharp/blunt laceration	29 (2.5%)	1 (0.6%)	
Avulsion injury	8 (0.7%)	2 (1.2%)	
Avulsion with partial skin defect	3 (0.3%)	3 (1.8%)	
PLT (×10⁹/L)	227.3 ± 53.4	282.2 ± 75.9	0.000
NEU% (%)	65.1 ± 9.9	77.3 ± 7.4	0.000
D-dimer (mg/L)	0.3 ± 0.4	0.8 ± 0.6	0.000
Hemoglobin (g/L)	138.3 ± 16.9	132.2 ± 19.8	0.000
Albumin (g/L)	39.1 ± 3.7	38.2 ± 3.4	0.002
Flap coverage			0.002
No	1152 (97.5%)	151 (92.6%)	
Yes	29 (2.5%)	12 (7.4%)	
Vascular crisis			0.000
None	1139 (96.4%)	135 (82.8%)	
Venous	2 (0.2%)	7 (4.3%)	
Arterial	40 (3.4%)	21 (12.9%)	

Values are *n* (%) or mean ± SD. *P* values are from Welch *t* tests or chi-square tests.

PLT, platelet count; NEU%, neutrophil percentage.

The same directional patterns were observed in the derivation cohort: patients with necrosis had higher PLT, NEU%, and D-dimer values, more vascular crises, and a lower proportion of procedures performed by specialist surgeons (Table 3).

**Table 3:** Complete characteristics by outcome in the derivation cohort.

Variable	Survival (*n* = 827)	Necrosis (*n* = 115)	*P* value
Sex			0.060
Male	651 (78.7%)	81 (70.4%)	
Female	176 (21.3%)	34 (29.6%)	
Age (years)	38.2 ± 14.0	40.0 ± 14.3	0.220
Ischemia time (hours)	5.0 ± 1.9	5.7 ± 2.4	0.003
Fingertip preservation			0.605
Isolated ice-bag cooling	3 (0.4%)	0 (0.0%)	
Clean dressing	656 (79.3%)	88 (76.5%)	
Room temperature/other	168 (20.3%)	27 (23.5%)	
Multiple/associated injury			0.729
No	753 (91.1%)	103 (89.6%)	
Yes	74 (8.9%)	12 (10.4%)	
Duration of surgery (hours)	2.8 ± 1.2	3.1 ± 1.4	0.041
Surgeon experience			0.000
Trainee	127 (15.4%)	70 (60.9%)	
Specialist	700 (84.6%)	45 (39.1%)	
Injured finger			0.651
Thumb	147 (17.8%)	18 (15.7%)	
Index	240 (29.0%)	35 (30.4%)	
Middle	169 (20.4%)	22 (19.1%)	
Ring	121 (14.6%)	13 (11.3%)	
Little	72 (8.7%)	11 (9.6%)	
Multiple digits	78 (9.4%)	16 (13.9%)	
Number of injured fingers			0.387
One	750 (90.7%)	99 (86.1%)	
Two	67 (8.1%)	14 (12.2%)	
Three	8 (1.0%)	2 (1.7%)	
Four	2 (0.2%)	0 (0.0%)	
Injury level			0.000
Free tissue block	67 (8.1%)	5 (4.3%)	
Distal to nail	125 (15.1%)	7 (6.1%)	
Middle nail level	191 (23.1%)	14 (12.2%)	
Nail-base level	262 (31.7%)	44 (38.3%)	
Distal interphalangeal joint	182 (22.0%)	45 (39.1%)	
Injury mechanism			0.095
Electric saw	51 (6.2%)	5 (4.3%)	
Crush	407 (49.2%)	57 (49.6%)	
Twisting	143 (17.3%)	30 (26.1%)	
Blunt impact	47 (5.7%)	1 (0.9%)	
Heat press	1 (0.1%)	0 (0.0%)	
Avulsion	13 (1.6%)	3 (2.6%)	
Sharp cut	165 (20.0%)	19 (16.5%)	
Smoking history			0.100
No	701 (84.8%)	90 (78.3%)	
Yes	126 (15.2%)	25 (21.7%)	
Hypertension			0.000
No	786 (95.0%)	97 (84.3%)	
Yes	41 (5.0%)	18 (15.7%)	
Diabetes			0.436
No	817 (98.8%)	112 (97.4%)	
Yes	10 (1.2%)	3 (2.6%)	
Vein graft			0.010
No	816 (98.7%)	109 (94.8%)	
Yes	11 (1.3%)	6 (5.2%)	
Degree of amputation			0.712
Incomplete	33 (4.0%)	6 (5.2%)	
Complete	794 (96.0%)	109 (94.8%)	
Bone injury			0.000
Comminuted fracture	320 (38.7%)	23 (20.0%)	
Intra-articular fracture	171 (20.7%)	40 (34.8%)	
Transverse fracture	336 (40.6%)	52 (45.2%)	
Nerve injury			0.000
Fingertip nerve injury	251 (30.4%)	16 (13.9%)	
Bilateral crush laceration	297 (35.9%)	47 (40.9%)	
Bilateral sharp laceration	159 (19.2%)	20 (17.4%)	
Bilateral avulsion injury	120 (14.5%)	32 (27.8%)	
Tendon injury			0.001
No tendon rupture	627 (75.8%)	69 (60.0%)	
Flexor/extensor zone I	192 (23.2%)	43 (37.4%)	
Flexor/extensor zone II	6 (0.7%)	1 (0.9%)	
Flexor/extensor zone V	2 (0.2%)	2 (1.7%)	
Number of arteries repaired			0.143
One	104 (12.6%)	12 (10.4%)	
Two	659 (79.7%)	88 (76.5%)	
Three or more	64 (7.7%)	15 (13.0%)	
Number of veins repaired			0.189
One	151 (18.3%)	15 (13.0%)	
Two	246 (29.7%)	37 (32.2%)	
Three	127 (15.4%)	25 (21.7%)	
Four	199 (24.1%)	21 (18.3%)	
Five or more	104 (12.6%)	17 (14.8%)	
Artery-to-vein repair ratio			0.498
1:0	9 (1.1%)	0 (0.0%)	
1:1	69 (8.3%)	8 (7.0%)	
1:2	47 (5.7%)	6 (5.2%)	
2:1	78 (9.4%)	7 (6.1%)	
2:2	232 (28.1%)	40 (34.8%)	
≥2:3	392 (47.4%)	54 (47.0%)	
Arterial injury			0.000
Fingertip artery transection	248 (30.0%)	17 (14.8%)	
Bilateral digital-artery crush injury	295 (35.7%)	43 (37.4%)	
Bilateral digital-artery sharp transection	160 (19.3%)	19 (16.5%)	
Bilateral digital-artery avulsion	117 (14.1%)	29 (25.2%)	
Avulsion with unilateral defect >1.5 cm	0 (0.0%)	2 (1.7%)	
Avulsion with bilateral defects >1.5 cm	7 (0.8%)	5 (4.3%)	
Nail-bed/dorsal skin injury			0.000
Nail-bed laceration	369 (44.6%)	28 (24.3%)	
Nail-bed and dorsal-skin injury	421 (50.9%)	80 (69.6%)	
Crush injury	8 (1.0%)	1 (0.9%)	
Sharp/blunt laceration	22 (2.7%)	1 (0.9%)	
Avulsion injury	5 (0.6%)	2 (1.7%)	
Avulsion with partial skin defect	2 (0.2%)	3 (2.6%)	
PLT (×10⁹/L)	229.4 ± 54.0	279.7 ± 78.9	0.000
NEU% (%)	64.8 ± 10.2	76.9 ± 7.5	0.000
D-dimer (mg/L)	0.3 ± 0.3	0.8 ± 0.6	0.000
Hemoglobin (g/L)	138.1 ± 17.0	133.2 ± 19.2	0.010
Albumin (g/L)	39.2 ± 3.7	38.4 ± 3.3	0.014
Flap coverage			0.000
No	810 (97.9%)	105 (91.3%)	
Yes	17 (2.1%)	10 (8.7%)	
Vascular crisis			0.000
None	796 (96.3%)	92 (80.0%)	
Venous	0 (0.0%)	6 (5.2%)	
Arterial	31 (3.7%)	17 (14.8%)	

Values are *n* (%) or mean ± SD. *P* values are from Welch *t* tests or chi-square tests.

PLT, platelet count; NEU%, neutrophil percentage.

### 3.3 Variable selection using LASSO with 10-fold cross-validation

Ten-fold cross-validated LASSO regression was used for variable reduction (Figure 2a). The minimum-deviance penalty was lambda.min = 0.005754182, whereas the more parsimonious one-standard-error penalty was lambda.1se = 0.01601135 (log [lambda.1se] = −4.134). The lambda.1se criterion was used and retained nine non-zero candidate variables: surgeon experience, injured finger, number of injured fingers, hypertension, tendon injury, PLT, NEU%, D-dimer, and vascular crisis (Figure 2b).

**Figure 2:**
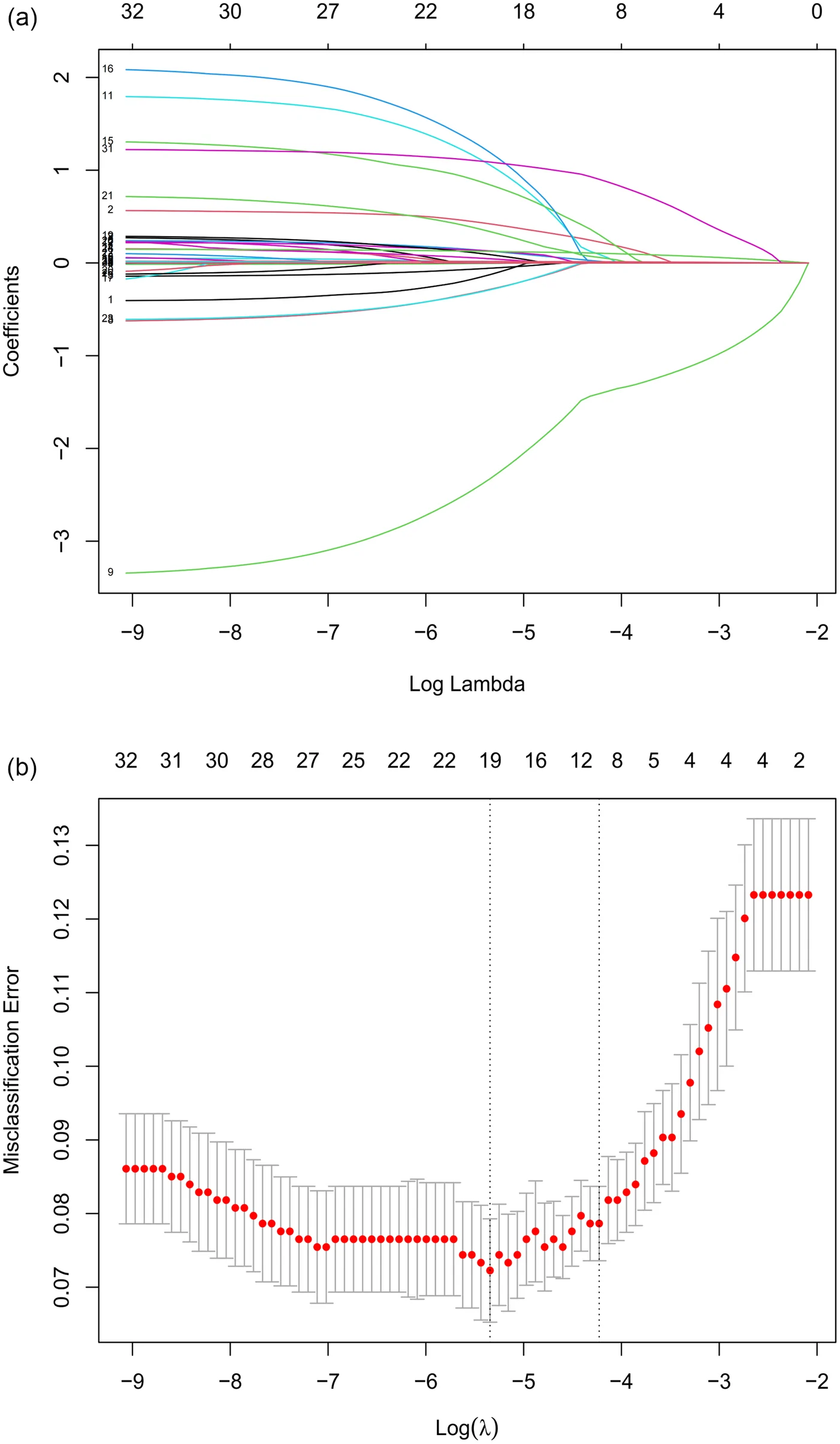
LASSO regression for predictor selection. **(a)** LASSO coefficient profiles of candidate variables. **(b)** Ten-fold cross-validation for selection of the optimal penalty parameter (*λ*). The *λ*.1se criterion was used to select nine candidate predictors.

### 3.4 Multivariable logistic regression model

The final six-predictor model is presented in Figure 3 and Table 4. Specialist surgeon status was associated with lower odds of necrosis (OR, 0.18; 95% CI, 0.10–0.32), whereas higher PLT (per 10 × 10⁹/L: OR, 1.20; 95% CI, 1.14–1.27), higher NEU% (per percentage point: OR, 1.17; 95% CI, 1.12–1.21), and higher D-dimer (per 1 mg/L: OR, 13.69; 95% CI, 6.21–30.18) were associated with higher odds. Hypertension (OR, 2.74; 95% CI, 1.04–7.24) and vascular crisis (OR, 2.79; 95% CI, 1.06–7.37) also remained independently associated with necrosis after adjustment.

**Figure 3:**
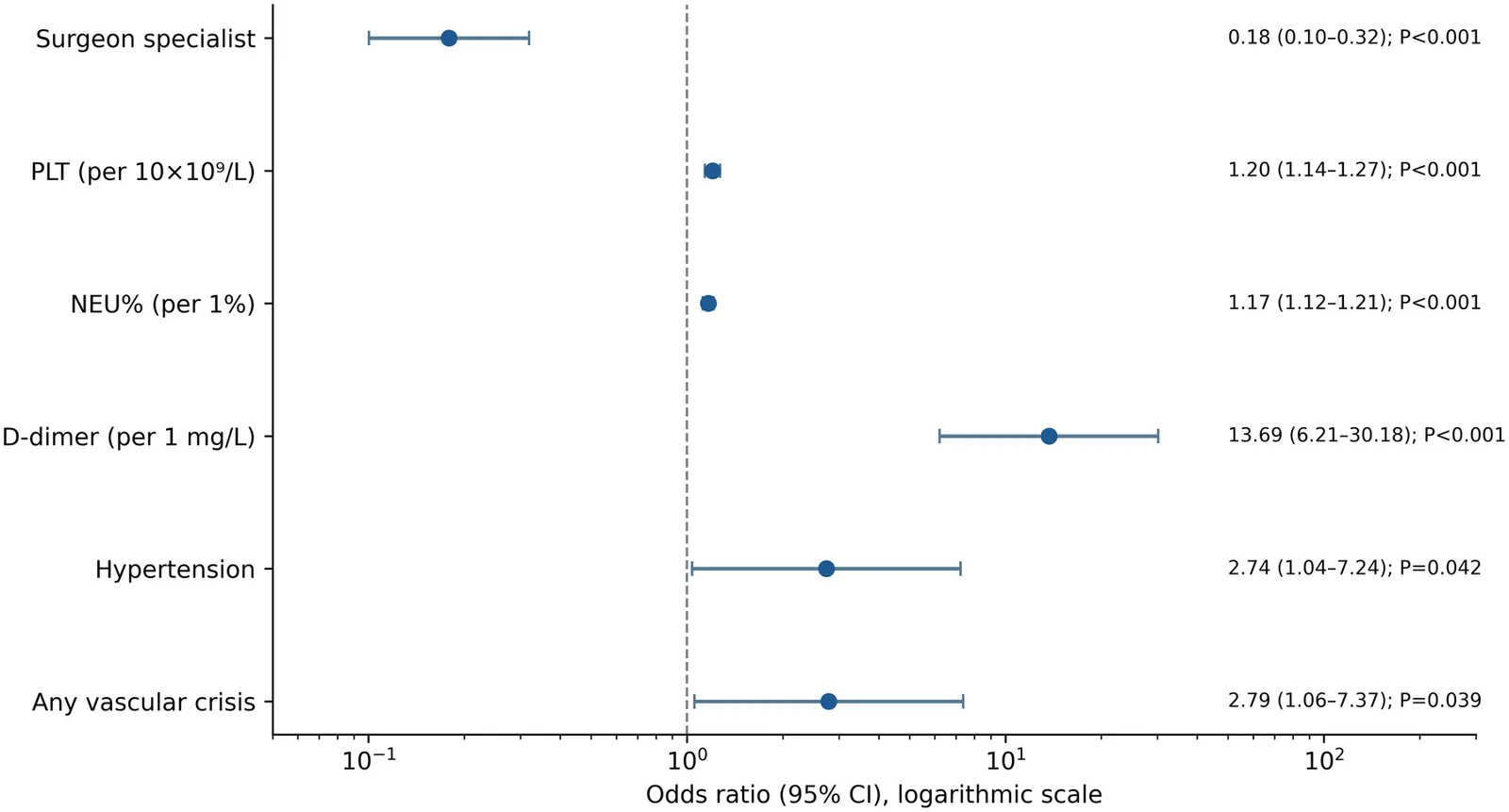
Forest plot of the multivariable logistic regression model. Points represent odds ratios (ORs), and horizontal lines represent 95% confidence intervals (CIs).

**Table 4:** Complete final logistic regression model.

Predictor	Coefficient (SE)	OR (95% CI)	*P* value; coding/unit
const	−17.7171 (SE 1.8022)	–	<0.001
Surgeon specialist (vs. trainee)	−1.7204 (SE 0.2954)	0.18 (0.10–0.32)	<0.001
PLT (per 10 × 10^9/L)	0.1843 (SE 0.0283)	1.20 (1.14–1.27)	<0.001
NEU% (per 1 percentage point)	0.1534 (SE 0.0194)	1.17 (1.12–1.21)	<0.001
D-dimer (per 1 mg/L)	2.6170 (SE 0.4032)	13.69 (6.21–30.18)	<0.001
Hypertension (yes vs. no)	1.0081 (SE 0.4956)	2.74 (1.04–7.24)	0.042; 1 = yes; 0 = no
Any vascular crisis (yes vs. no)	1.0255 (SE 0.4958)	2.79 (1.06–7.37)	0.039; 1 = arterial/venous; 0 = none

logit(p) = −17.7171–1.7204(specialist) + 0.0184332(PLT) + 0.153379(NEU%) + 2.617017(D-dimer) + 1.008071(hypertension) + 1.025548(any vascular crisis).

### 3.5 Nomogram for early-postoperative risk estimation

A nomogram was constructed from the complete multivariable model (Figure 4). The linear predictor was: −17.7171−1.7204 × specialist surgeon + 0.0184332 × PLT + 0.153379 × NEU% + 2.617017 × D-dimer + 1.008071 × hypertension + 1.025548 × any vascular crisis. Binary indicators were coded 1 when present and 0 otherwise. Predicted probability was calculated as 1/ [1 + exp(−linear predictor)]. The nomogram should be applied only after postoperative laboratory results obtained on days 3–5 and vascular-crisis status are available.

**Figure 4:**
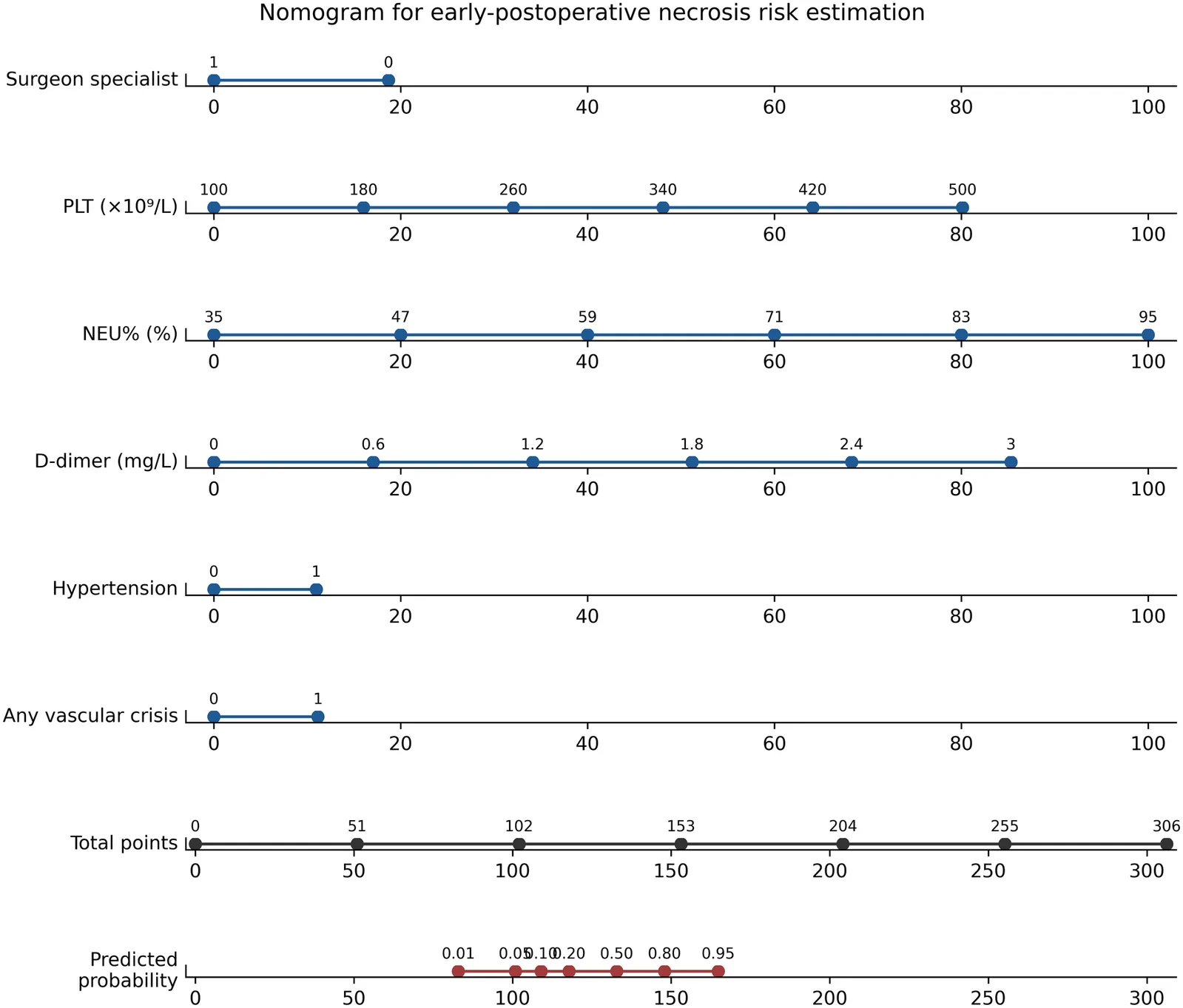
Nomogram for predicting necrosis after fingertip replantation. The nomogram was developed using six predictors identified by multivariable logistic regression.

### 3.6 Model performance and split-sample internal validation

The derivation-cohort AUC was 0.953 (95% CI, 0.935–0.968), and the internal-validation AUC was 0.974 (95% CI, 0.958–0.987) (Figures 5a, b). Brier scores were 0.051 and 0.042, respectively. Apparent calibration in the derivation cohort had an intercept of approximately 0 and a slope of approximately 1, as expected when assessed in the model-development data. In the held-out cohort, the calibration intercept was −0.338 and the slope was 0.759, suggesting modest overall overprediction and predictions that were somewhat too extreme. Hosmer–Lemeshow tests did not detect statistically significant lack of fit in either the derivation cohort (*P* = 0.752) or the internal validation cohort (*P* = 0.729). However, nonsignificant Hosmer–Lemeshow results do not establish good or excellent calibration and were interpreted together with the calibration plot, intercept, slope, and Brier score (Figures 5c, d). Decision-curve analysis suggested potential net benefit over treat-none within portions of the examined threshold range; it does not establish clinical utility at a specific threshold, which requires prospective external evaluation (Figures 5e, f).

**Figure 5:**
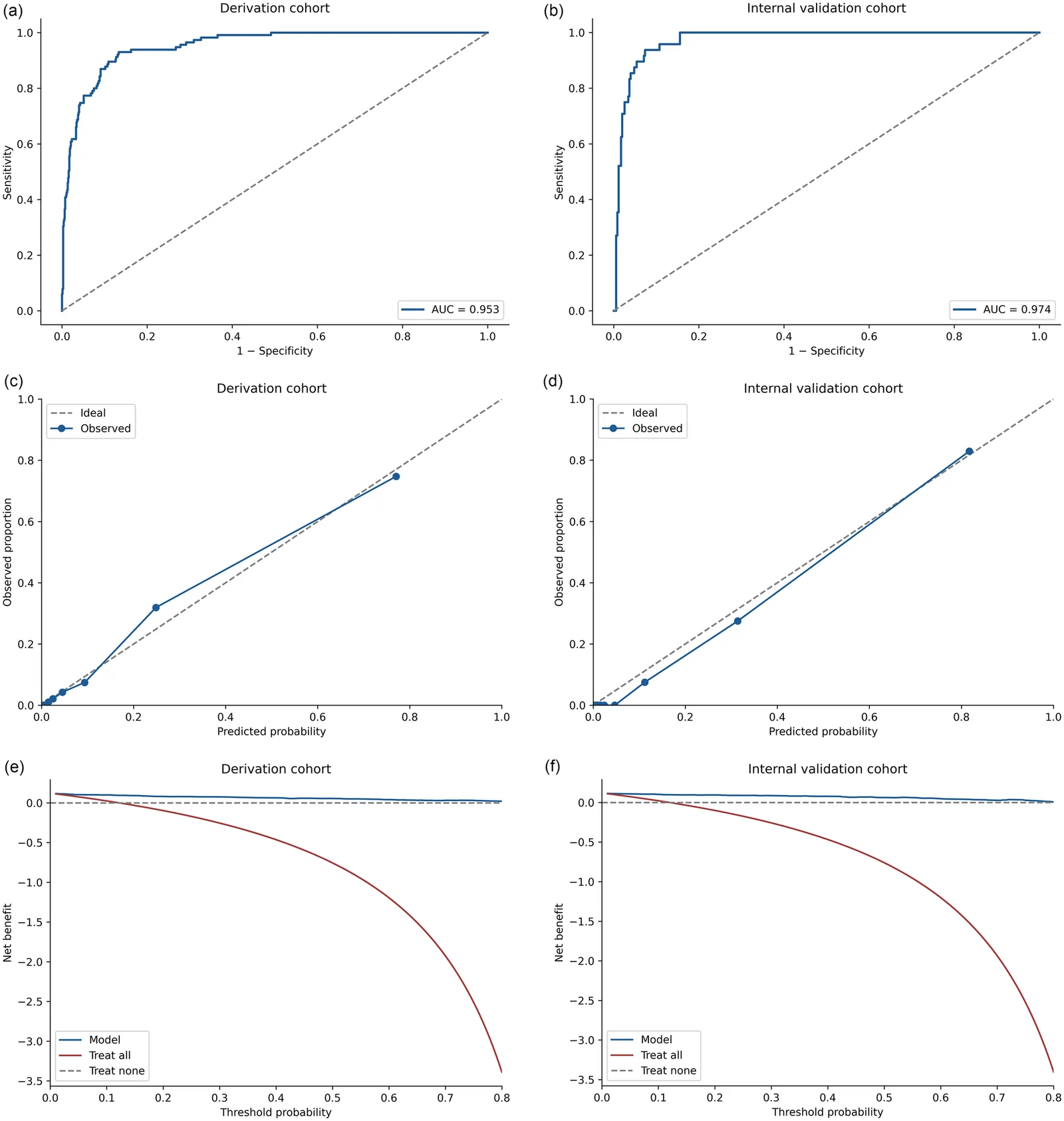
Performance of the prediction model. **(a,b)** ROC curves in the derivation and internal validation cohorts, respectively. **(c,d)** Calibration curves in the derivation and internal validation cohorts, respectively. **(e,f)** Decision curve analyses in the derivation and internal validation cohorts, respectively.

## 4 Discussion

We developed a six-predictor model for necrosis after fingertip replantation using information available during the early postoperative course. Unlike models intended for preoperative counseling, this nomogram incorporates laboratory measurements obtained 3–5 days after surgery and vascular-crisis status. Its potential value lies in combining distal-fingertip-specific procedural information with routinely measured inflammatory and coagulation markers. High AUC values were observed in the split-sample cohorts; however, the validation calibration slope of 0.759 suggests some overfitting, and the findings should be interpreted cautiously until externally validated.

Prior studies have reported prediction models and individual risk factors for digit-replantation failure (5–8). The present nomogram is therefore an incremental rather than first-in-field contribution. It is distinguished by its focus on distal fingertip replantation and by incorporation of early-postoperative PLT, NEU%, D-dimer, and vascular-crisis status. These features may support intensified monitoring once postoperative data are available, but they also restrict portability and prevent preoperative use.

Specialist surgeon status was strongly associated with lower necrosis odds, plausibly reflecting greater experience with small-vessel anastomosis and venous identification (9). Hypertension and vascular crisis had directionally positive adjusted associations but wide confidence intervals crossing unity. Vascular crisis is also a post-surgical intermediate event on the pathway to necrosis; its inclusion improves contemporaneous risk estimation but limits causal interpretation and means that the nomogram should not be described as a baseline prognostic model.

Higher PLT and D-dimer were associated with increased necrosis risk, consistent with thrombotic and hypercoagulable mechanisms. Wang et al. (6) reported that postoperative D-dimer on day 1 independently predicted replantation necrosis. Our measurements were obtained 3–5 days after surgery, a period that overlaps with the occurrence of vascular compromise. These markers may therefore capture both predisposition and evolving postoperative pathology rather than purely pre-existing risk.

Postoperative NEU% was higher in the necrosis group than in the survival group, consistent with the positive adjusted coefficient (OR, 1.17 per percentage point). Inflammation can promote thrombosis through endothelial activation, neutrophil recruitment, platelet adhesion, and a hypercoagulable state (10, 11). This association is biologically plausible but should not be interpreted as causal because NEU% was measured after surgery and may reflect tissue injury, infection, or an evolving vascular event. Mechanistic and longitudinal studies are needed.

This study has several limitations. It was retrospective and conducted at a single high-volume center. Only split-sample internal validation was performed, and the validation calibration slope indicated some overfitting; multicenter external validation and model updating are required. Although source verification resolved the single spreadsheet transcription error and no analyzed values were missing, retrospective data collection remains susceptible to undocumented measurement or recording error. The data file contained one patient-level record per patient despite 1,497 replanted fingertips; thus finger-specific outcomes and within-patient heterogeneity among multidigit injuries could not be modeled. Variable selection followed by ordinary logistic regression may introduce optimism. The inclusion of postoperative laboratory values and vascular crisis restricts the model to the early postoperative setting, and vascular crisis may be an intermediate event. Finally, the definition of specialist experience (>50 replantations) and case mix at this high-volume center may not transport to lower-volume settings.

## 5 Conclusion

This nomogram estimates necrosis risk after fingertip replantation using six clinical, procedural, and early-postoperative predictors (12–18). Although high AUC values were observed in split-sample internal validation, calibration shrinkage was present and no external validation has been performed. The model should therefore not be considered ready for routine clinical decision-making; if evaluated prospectively, it should be treated only as an adjunct during the early postoperative course. Future work should externally validate and, if necessary, recalibrate the model in diverse settings and investigate the mechanisms linking inflammatory and coagulation markers with necrosis.

## Data Availability

The raw data supporting the conclusions of this article will be made available by the authors, without undue reservation.
